# Supplementary material for: The Role of SCL Isoforms in Embryonic Hematopoiesis
Source: Int J Mol Sci. 2023 Mar 29;24(7):6427. doi: 10.3390/ijms24076427 (PMC10094407; doi:10.3390/ijms24076427)
Supplement: Supplementary file 1 [file ijms-24-06427-s001.zip › ijms-1944225-supplementary.pdf]

SUPPLEMENTARY FIGURE

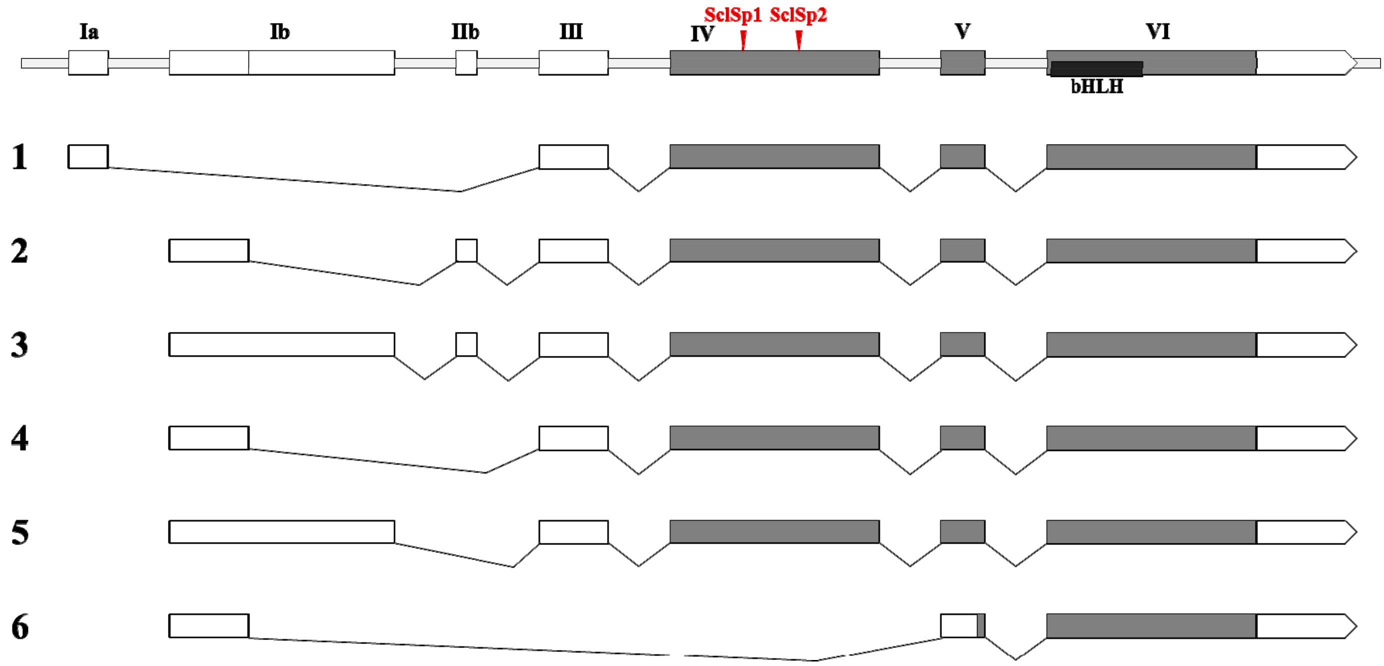

Figure S1. Isoforms of mouse *SCL* mRNA

Isoforms of mouse *SCL* mRNA which had been identified by cDNA cloning and sequencing. All of the alternatively spliced mRNA isoforms include exon IV, V and VI encoding a 329 a.a. full length SCL-L protein, except the sixth one without exon IV and encoding a short form SCL-S protein of 154 a.a. translationally initiated in exon V. The locations of the two candidates of protospacer with PAM are marked by arrowheads. The coding region is marked as gray bars and the bHLH domain is indicated by a black bar.

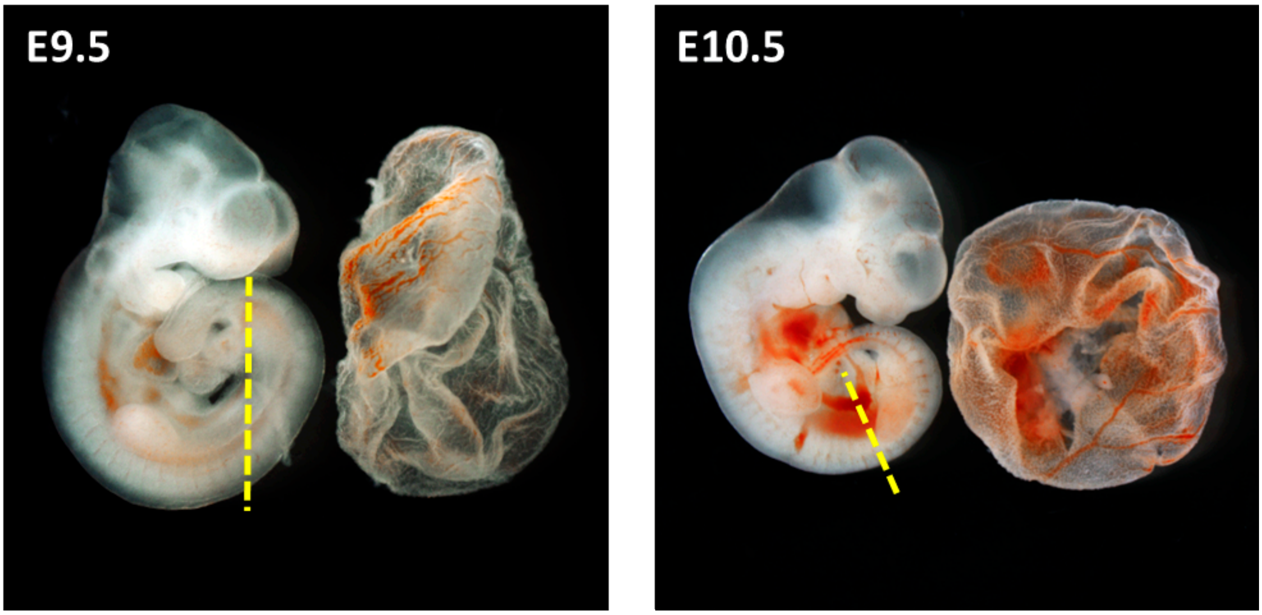

Figure S2. Cross sections of embryos for immune staining

The cutting angles of the E9.5 and E10.5 sections across aorta were indicated by yellow dashed lines.

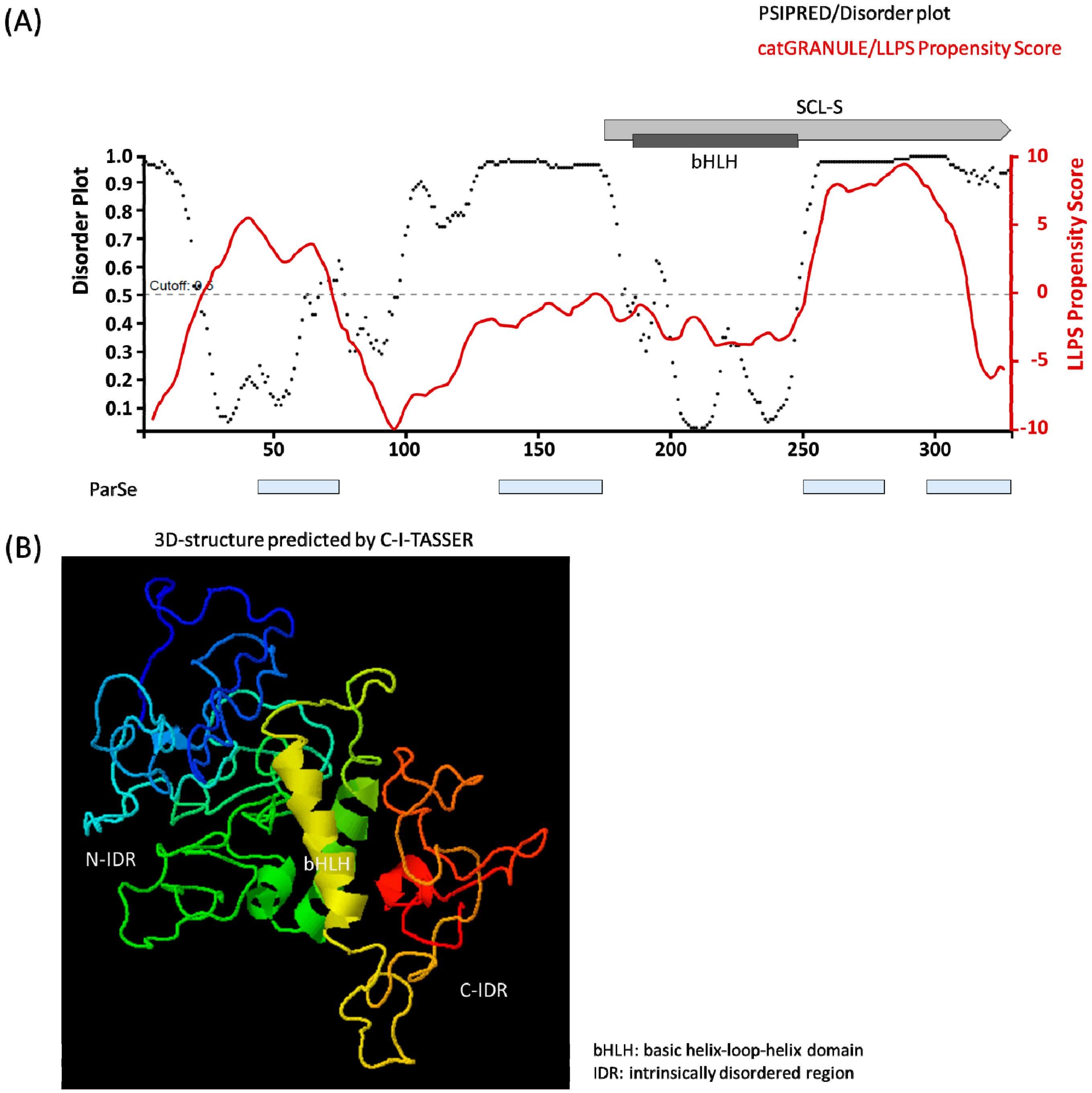

Figure S3. Predictions of the tendency of structural disorder and phase separation of the mouse SCL protein (A) The propensity score of disorder (cut-off = 0.5) and liquid-liquid phase separation (LLPS, cut-off = 0) were predicted by PSIPRED and catGRANULE, respectively, relative to the amino acid sequence of mouse SCL-L protein. The corresponding regions of SCL-S and bHLH domain are indicated. (B) The 3D-structure of mouse full length SCL predicted by C-I-TASSER is illustrated.
